# Supplementary figures and images for: Inositol-requiring enzyme 1α/X-box protein 1 pathway expression is impaired in pediatric cholestatic liver disease explants
Source: PLoS One. 2022 Dec 15;17(12):e0279016. doi: 10.1371/journal.pone.0279016 (PMC9754178; doi:10.1371/journal.pone.0279016)

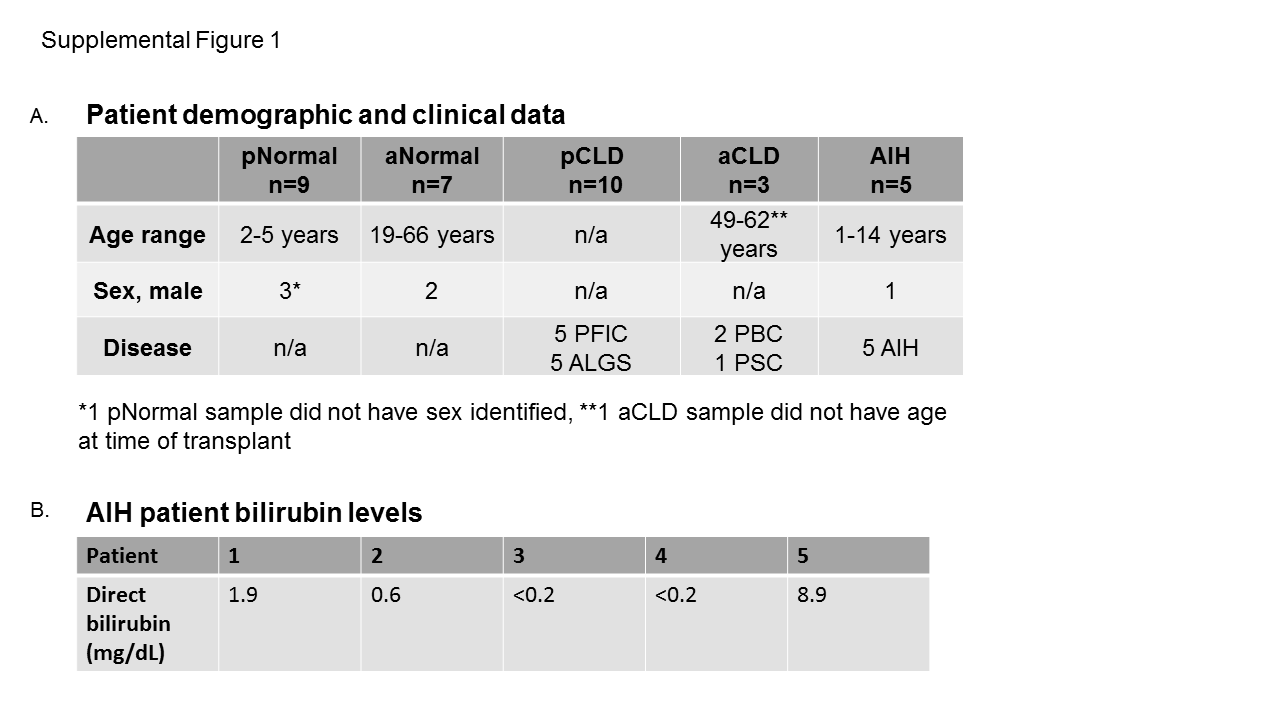

Supplement: S1 Fig — A) Demographic data of 34 human liver explant samples. B) Direct bilirubin level (mg/dL) of autoimmune hepatitis (AIH) patients (n = 5) on the day prior to liver transplantation. pNormal = pediatric normal liver, aNormal = adult normal liver, pCLD = pediatric cholestatic liver disease, aCLD = adult cholestatic liver disease, AIH = autoimmune hepatitis, mos = months. (TIF) [file pone.0279016.s001.tif]

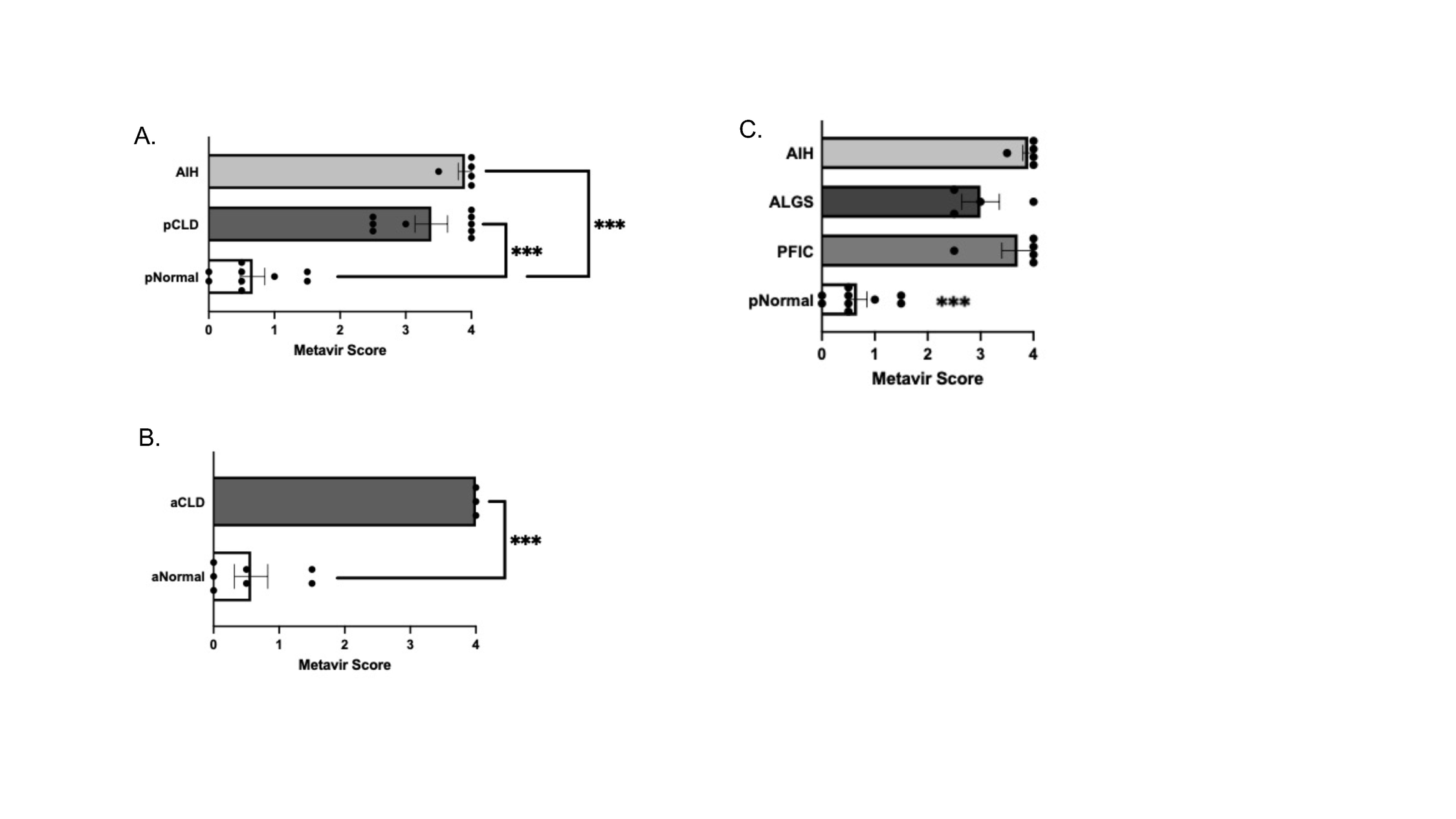

Supplement: S2 Fig — A) Pediatric cholestatic liver disease (pCLD) (n = 9) and autoimmune hepatitis (AIH) livers (n = 4) demonstrated significantly more fibrosis than pediatric normal samples(pNormal) (n = 9) (***p<0.001). B) Adult cholestatic liver disease (aCLD) livers (n = 3) had significantly more fibrosis than adult normal (aNormal) samples (n = 7) (***p<0.001). C) Alagille (ALGS) (n = 4) and progressive familial intrahepatic cholestasis (PFIC) samples (n = 5) had significantly more fibrosis than pNormal (n = 9) and had similar METAVIR scores to AIH samples (n = 4) (***p<0.001). (TIF) [file pone.0279016.s002.tif]

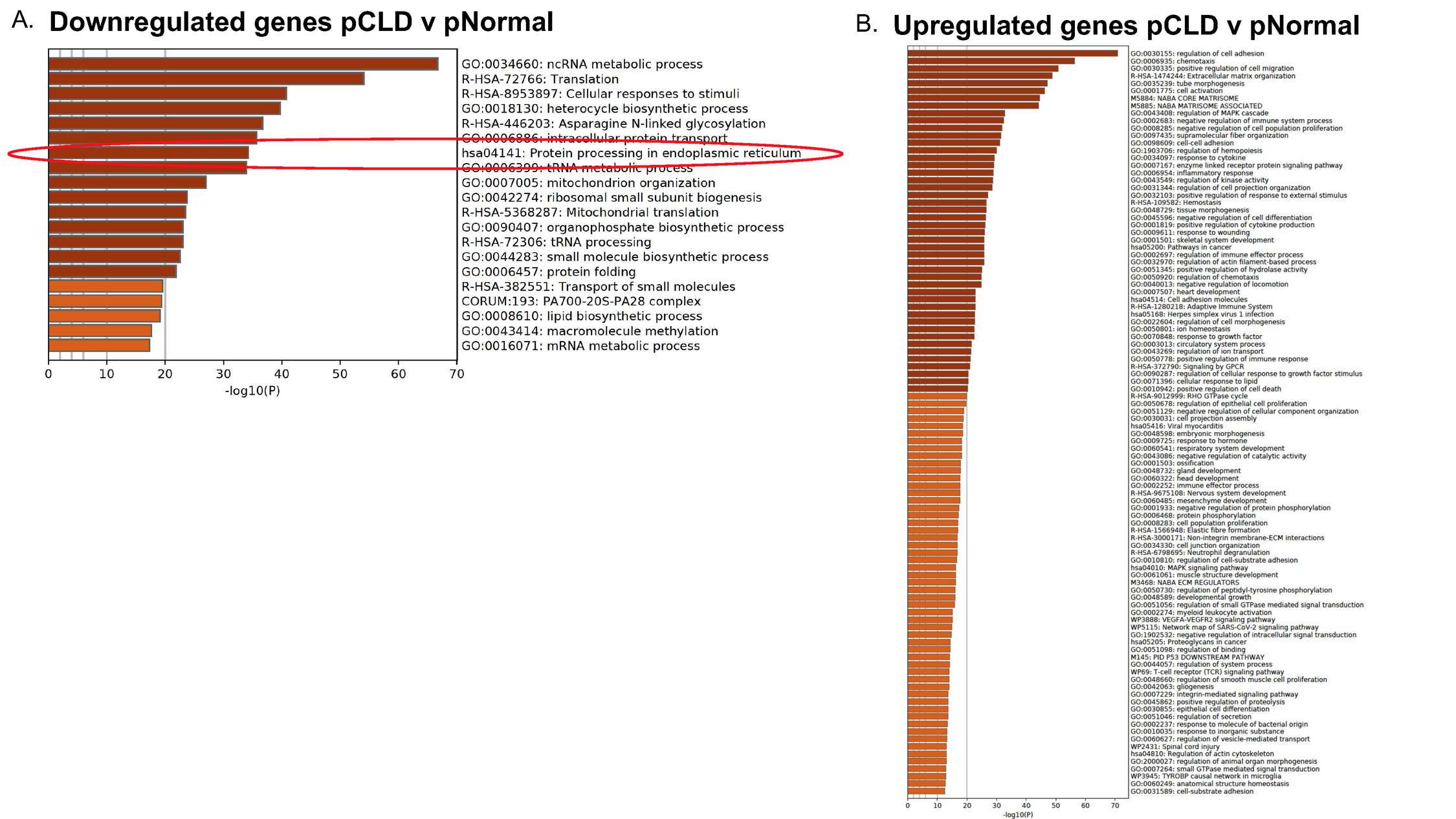

Supplement: S3 Fig — A) Utilizing all significantly different downregulated genes in pCLD for pathway analysis, the KEGG pathway hsa04141 “protein processing in endoplasmic reticulum” (circled in red) was among the top 10 differentially expressed pathways (-log10(P) = 34.28). B) Utilizing the top significantly upregulated genes in pCLD for pathway analysis KEGG pathway hsa04141 “protein processing in endoplasmic reticulum” was not differentially expressed. (TIF) [file pone.0279016.s003.tif]

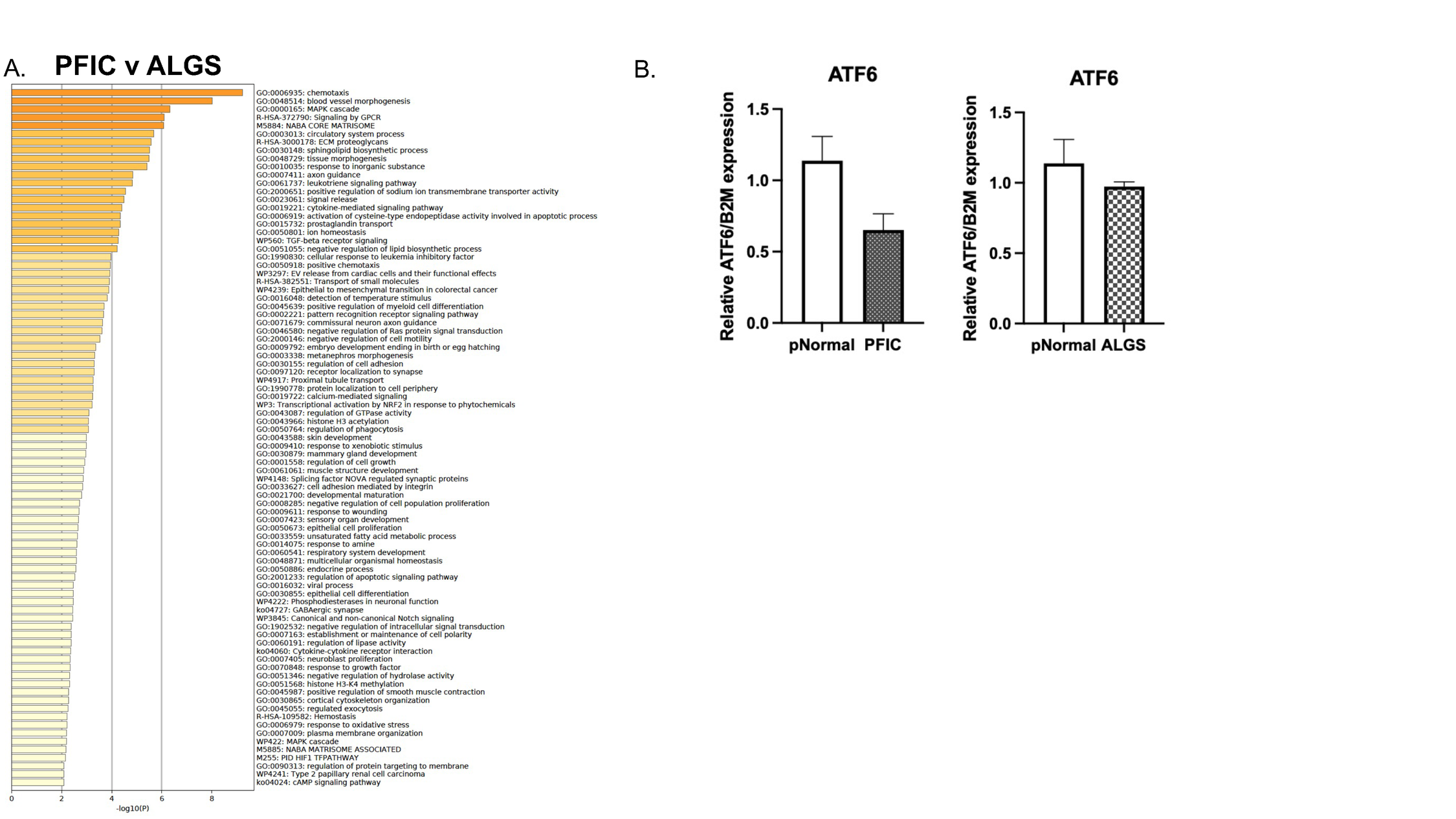

Supplement: S4 Fig — A) The top 100 pathways enriched in differentially expressed genes. B) There was no decreased ATF6 gene expression in PFIC (n = 5) and ALGS (n = 5) livers compared to pNormal livers (n = 9) (p = 0.07 and p = 0.5 respectively). (TIF) [file pone.0279016.s004.tif]

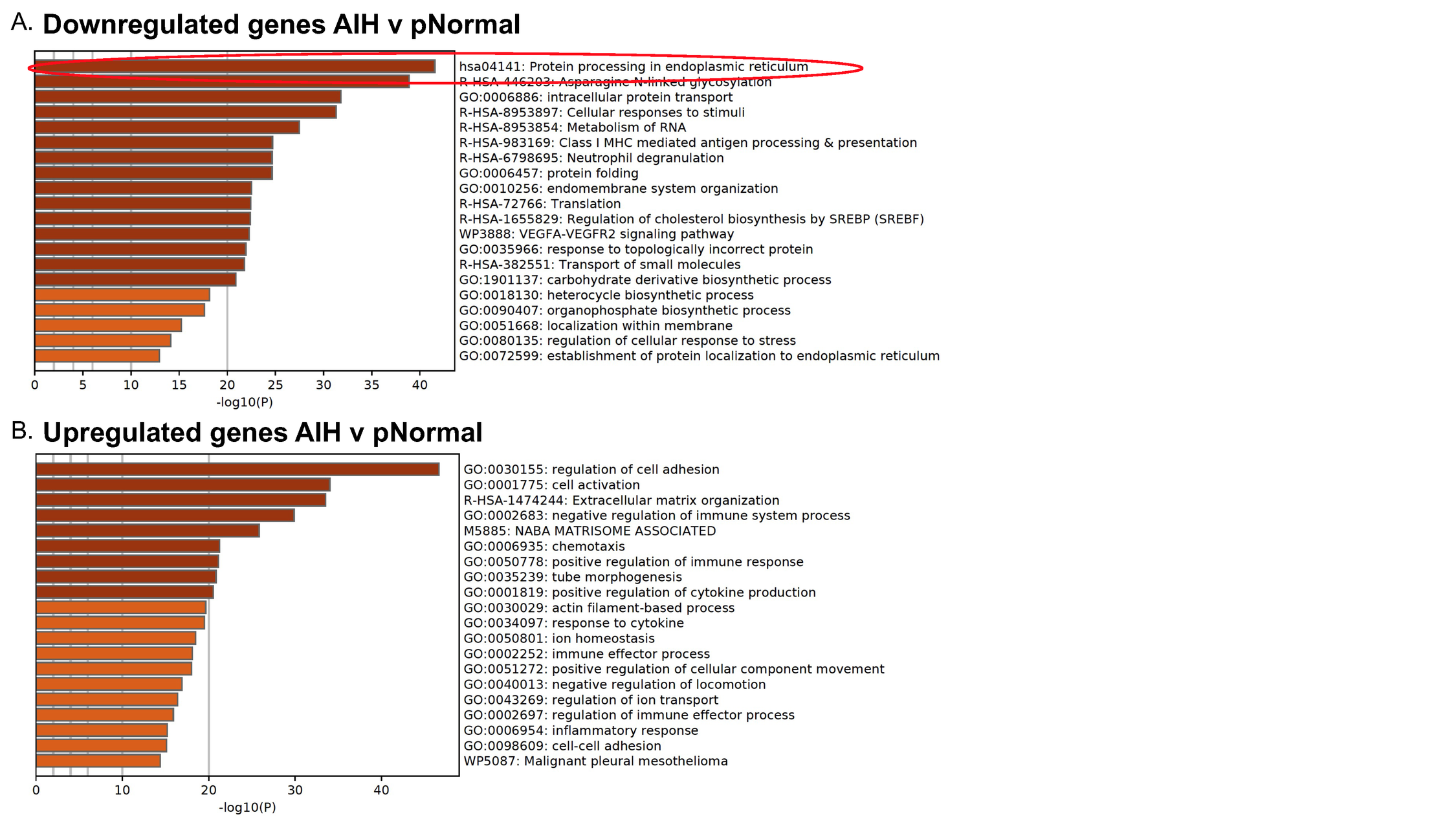

Supplement: S5 Fig — A) Utilizing all significantly different downregulated genes in AIH samples for Metascape pathway analysis, the KEGG pathway hsa04141 “protein processing it endoplasmic reticulum” (circled in red) was the top differentially expressed pathway (-log10(P) = 41.54). B) Utilizing all significantly different upregulated genes in AIH samples for pathway analysis. The top 20 differentially expressed pathways are shown. (TIF) [file pone.0279016.s005.tif]

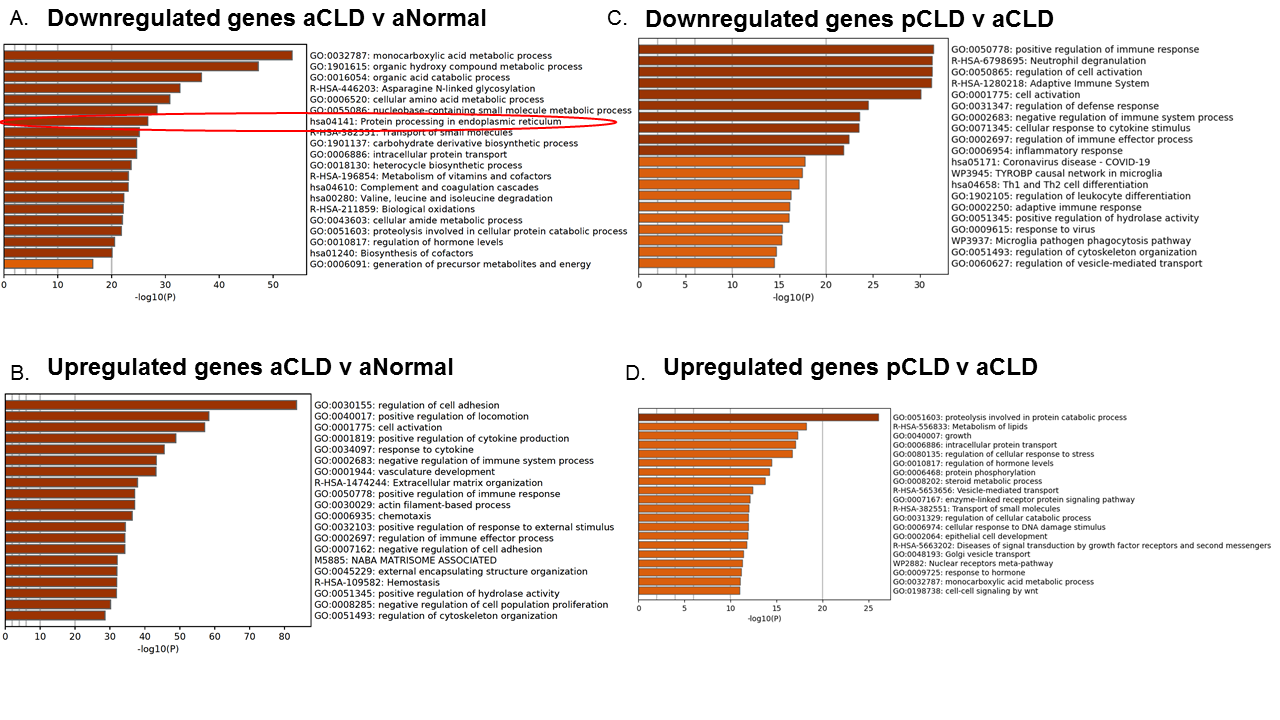

Supplement: S6 Fig — A) Utilizing all significantly different downregulated genes in aCLD samples (compared to aNormal) for Metascape pathway analysis, the KEGG pathway hsa04141 “protein processing it endoplasmic reticulum” (circled in red) was the top 10 differentially expressed pathway (-log10(P) = 26.74). B) Utilizing all significantly different upregulated genes in aCLD samples (compared to aNormal) for Metascape pathway analysis, KEGG pathway hsa04141 “protein processing in endoplasmic reticulum” was not in the top 100 pathways enriched in these genes. The top 20 differentially expressed pathways are shown. C) Utilizing all significantly different downregulated genes in pCLD compared to aCLD samples for pathway analysis. The top 20 differentially expressed pathways are shown. D) Utilizing all significantly different upregulated genes in pCLD samples compared to aCLD for pathway analysis. The top 20 differentially expressed pathways are shown. (TIF) [file pone.0279016.s006.tif]

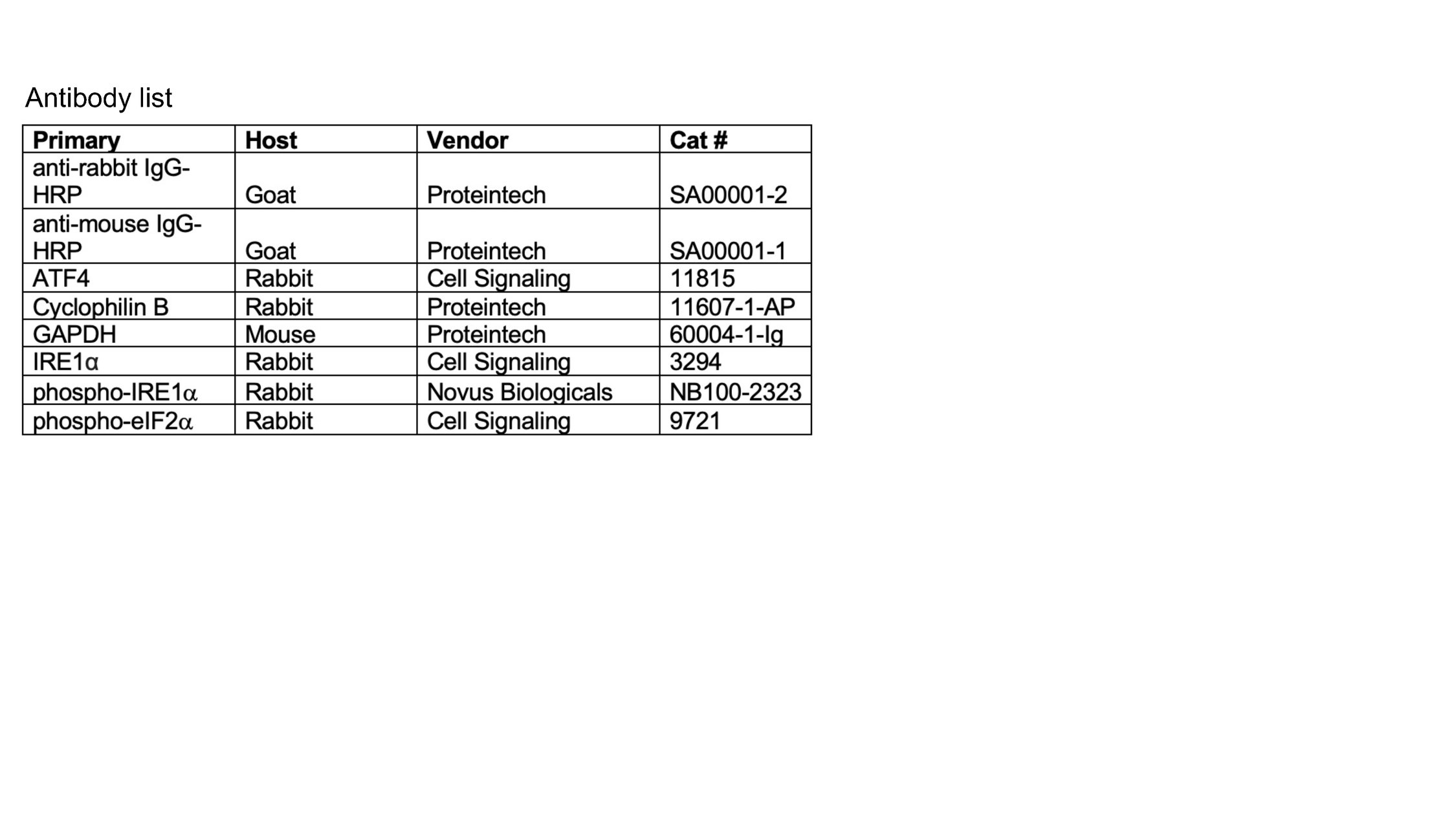

Supplement: S1 Table — (TIF) [file pone.0279016.s007.tif]

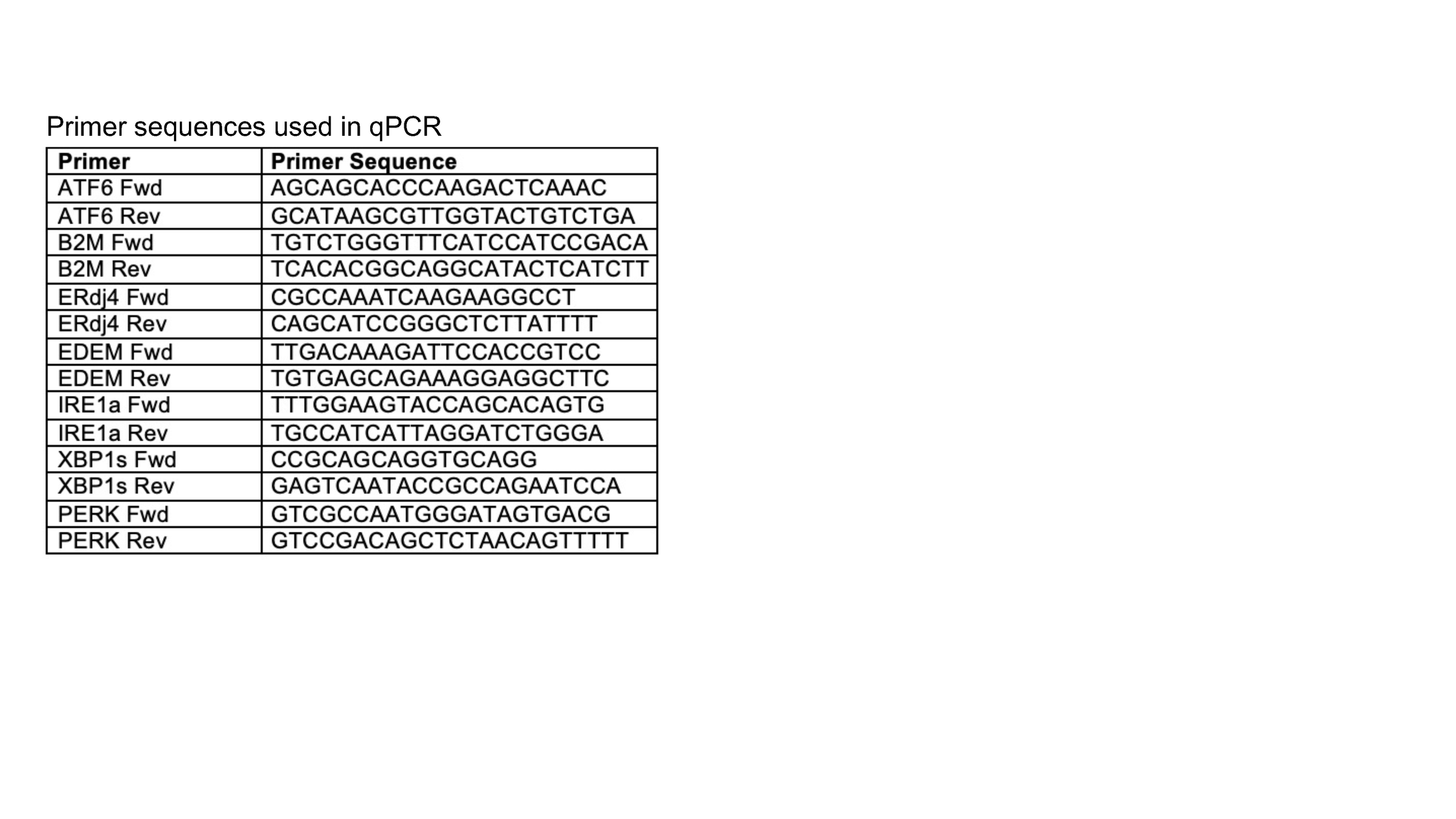

Supplement: S2 Table — (TIF) [file pone.0279016.s008.tif]
