# Supplementary material for: Inositol-requiring enzyme 1α/X-box protein 1 pathway expression is impaired in pediatric cholestatic liver disease explants
Source: PLoS One. 2022 Dec 15;17(12):e0279016. doi: 10.1371/journal.pone.0279016 (PMC9754178; doi:10.1371/journal.pone.0279016)

Original blot from figure 2C
p-IRE1α

'+' = positive control for sample

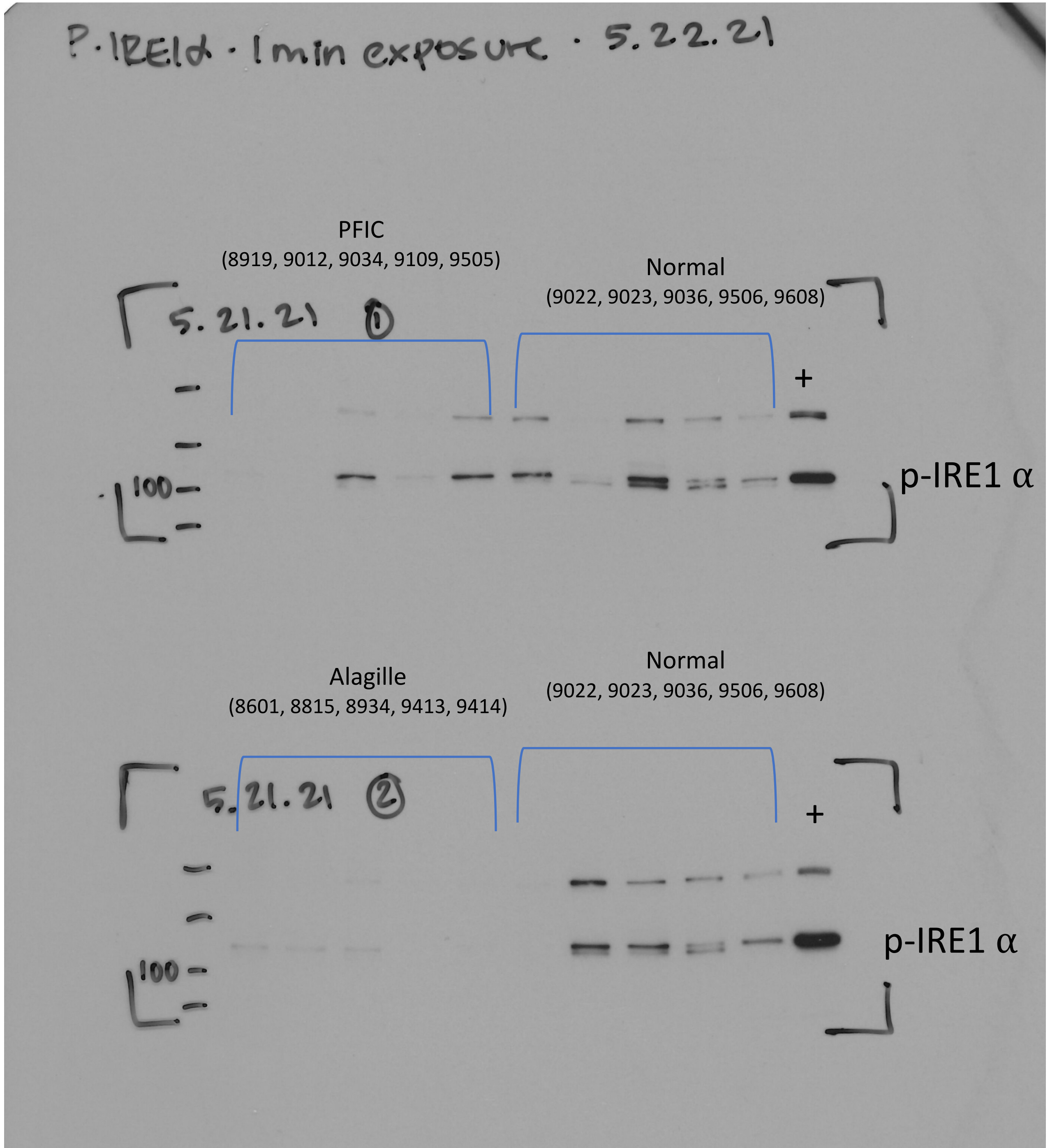

Original blot from figure 2C
cyclophilin B

'+' = positive control sample

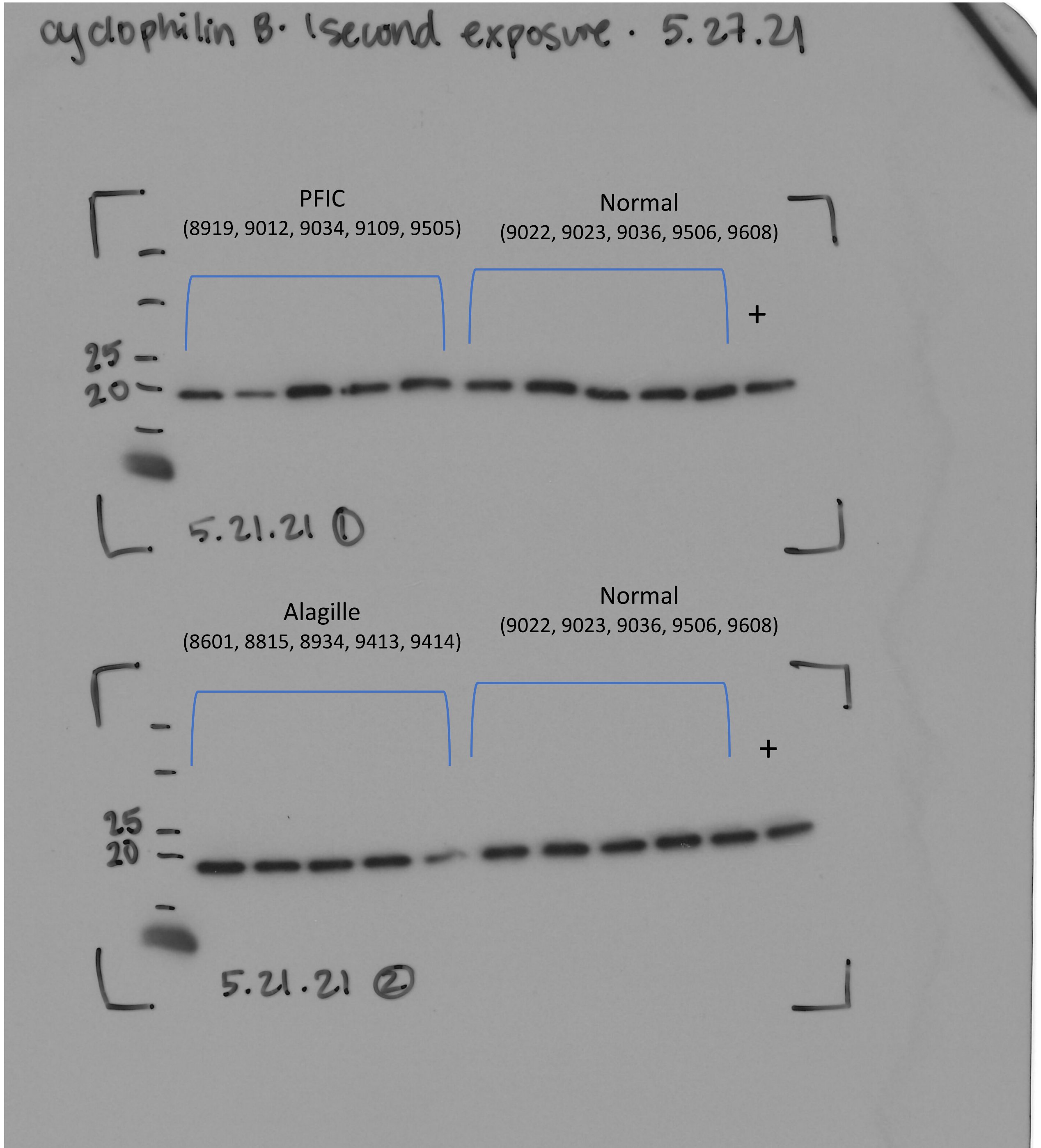

Original blot from figure 3A and 5D and 6D for p-eIF2α

(Ped. Cholestatic = pediatric cholestatic liver disease samples; Ped. Normal = pediatric normal samples; Adult Chol. = adult cholestatic liver disease samples; AIH = autoimmune hepatitis samples; ‘+’ = positive control for antibody

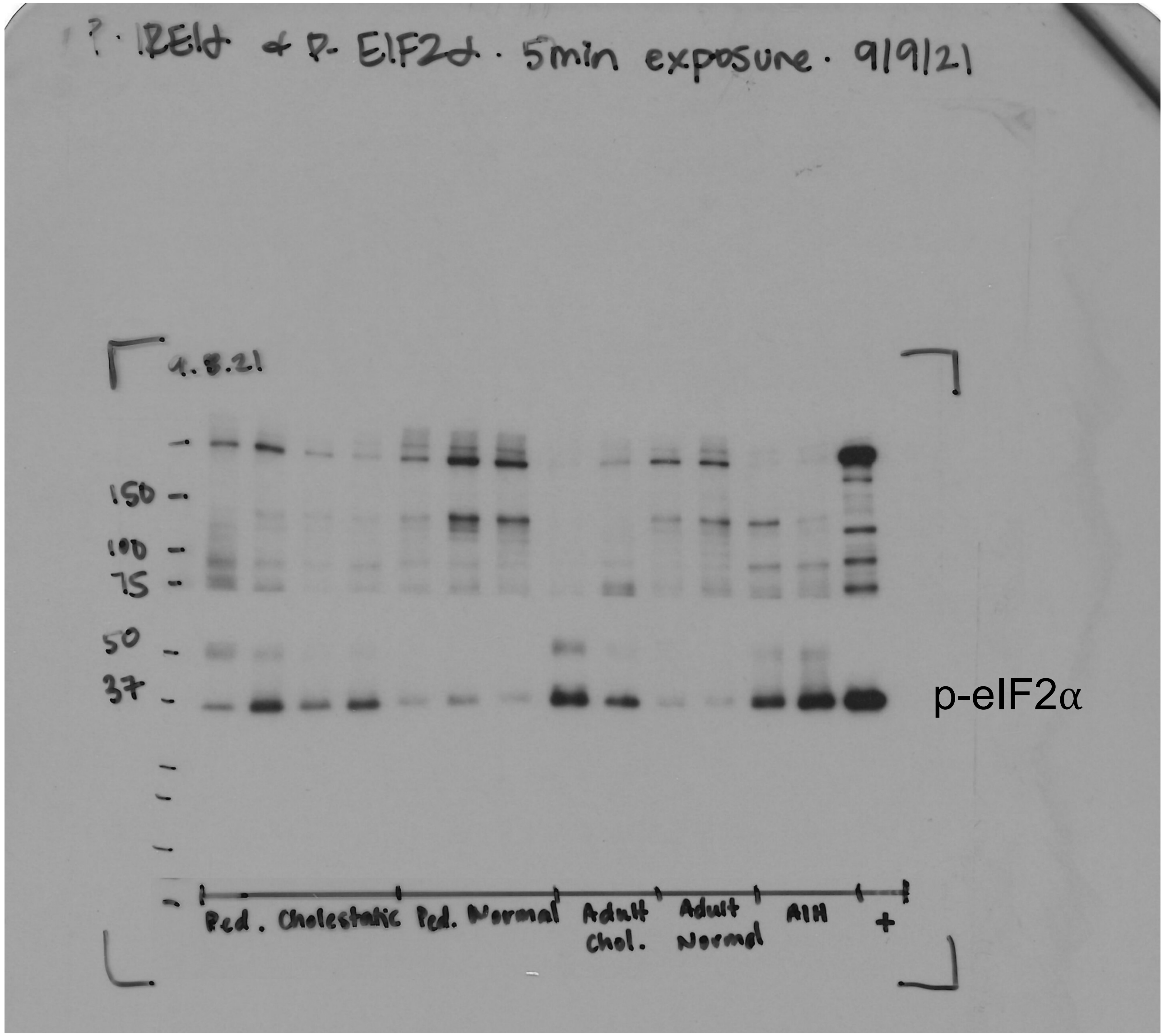

Original blot from figure 3A and 5D and 6D for ATF4

(Ped. Cholestatic = pediatric cholestatic liver disease samples; Ped. Normal = pediatric normal samples; Adult Chol. = adult cholestatic liver disease samples; AIH = autoimmune hepatitis samples; '+' = positive control for antibody

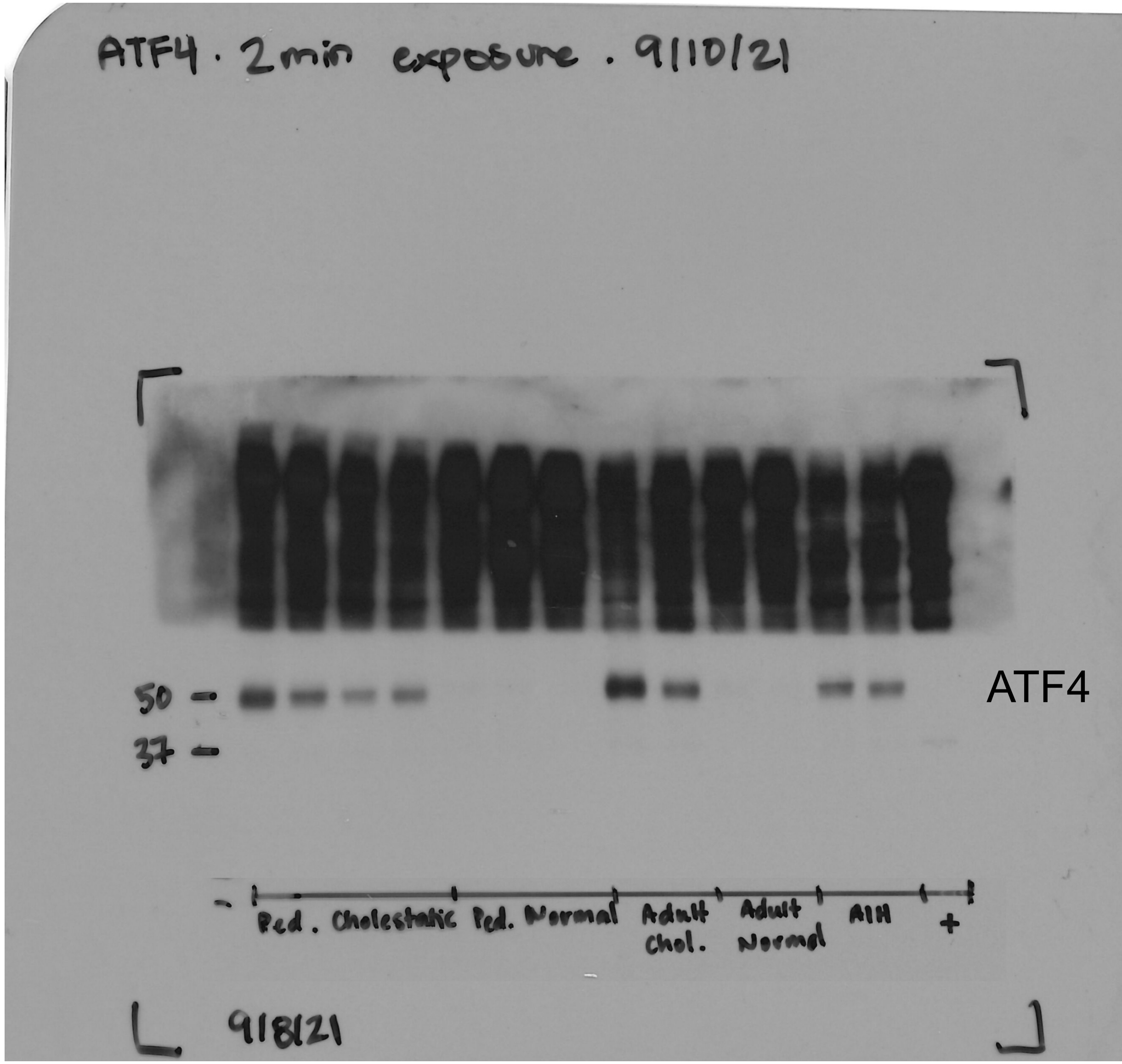

Original blot from figure 3A and 5D and 6D for cyclophilin

(Ped. Cholestatic = pediatric cholestatic liver disease samples; Ped. Normal = pediatric normal samples; Adult Chol. = adult cholestatic liver disease samples; AIH = autoimmune hepatitis samples;
'+' = positive control for antibody

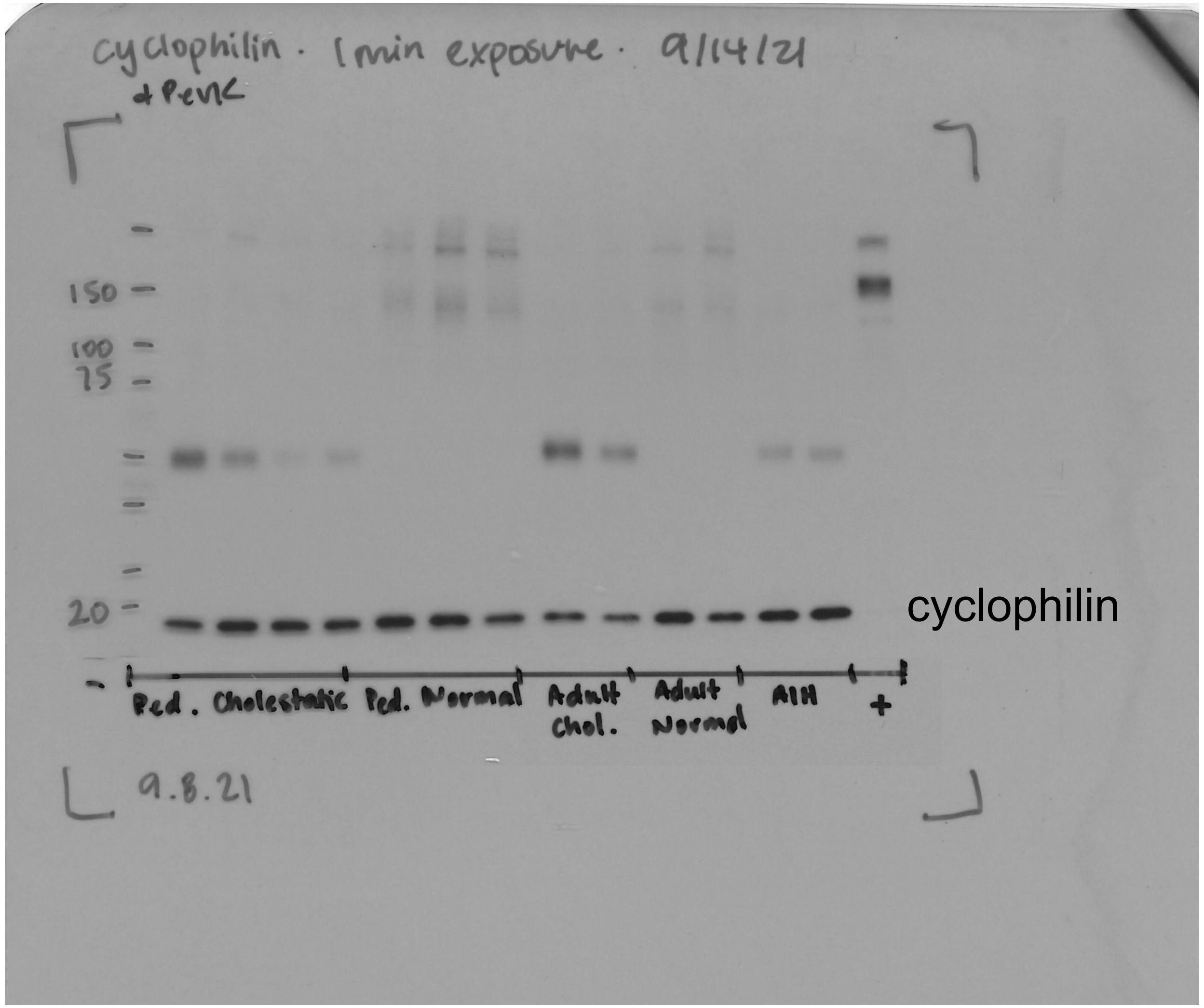

Original blot from figure 3B for p-eIF2α

'+' = positive control sample

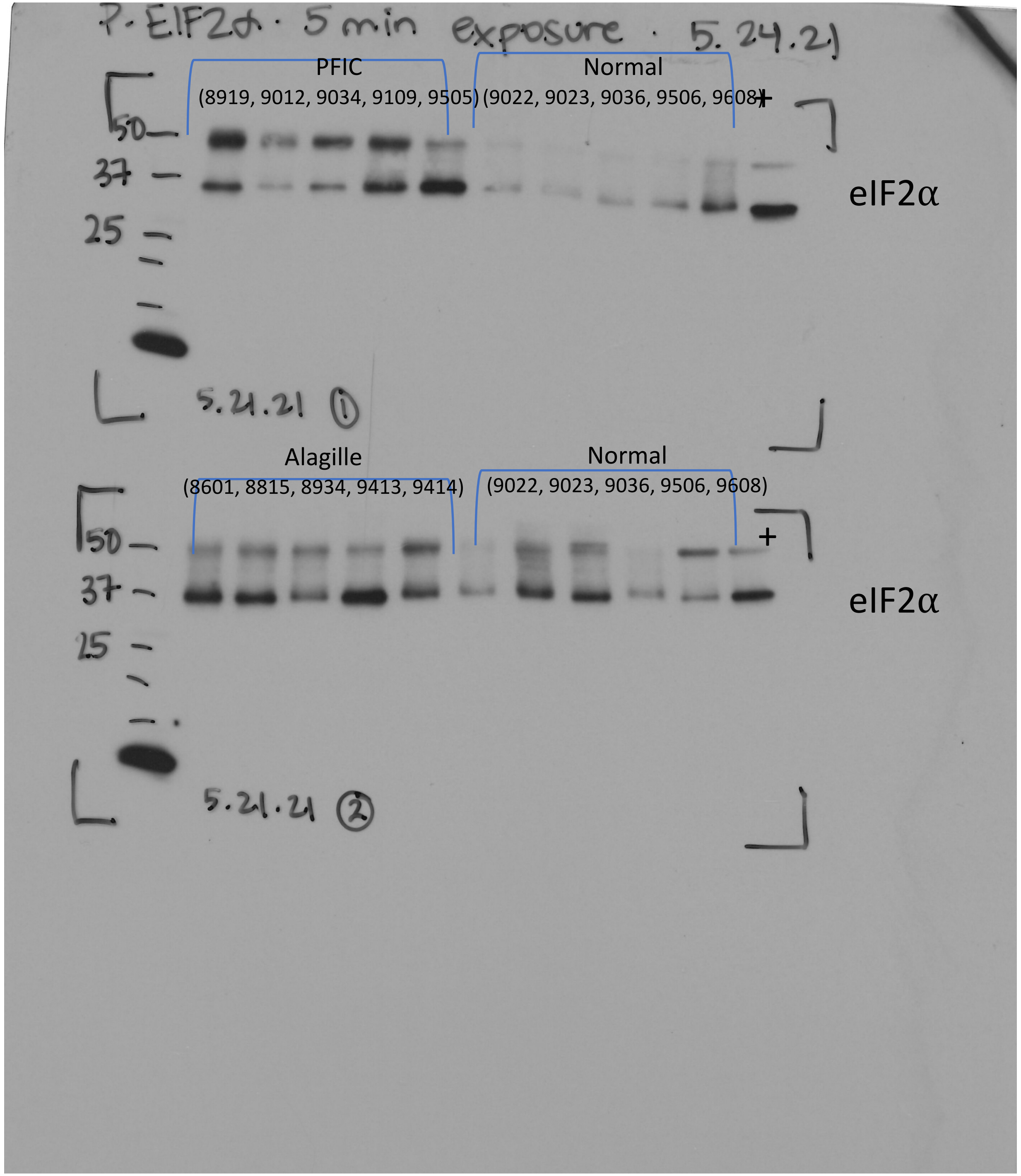

Original blot from figure 3B for ATF4

'+' = positive control sample

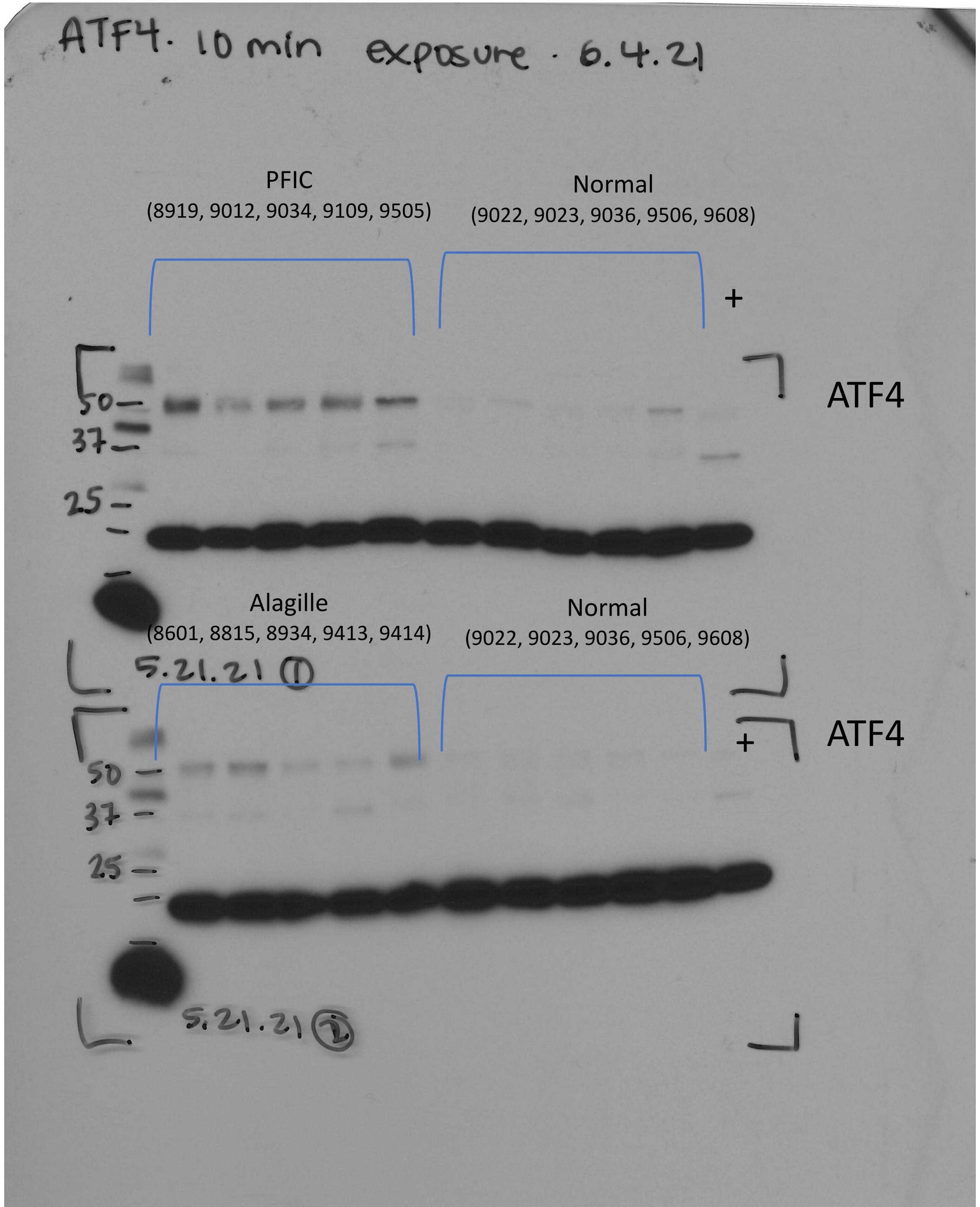

Original blot from figure 3B for cyclophilin

'+' = positive control sample

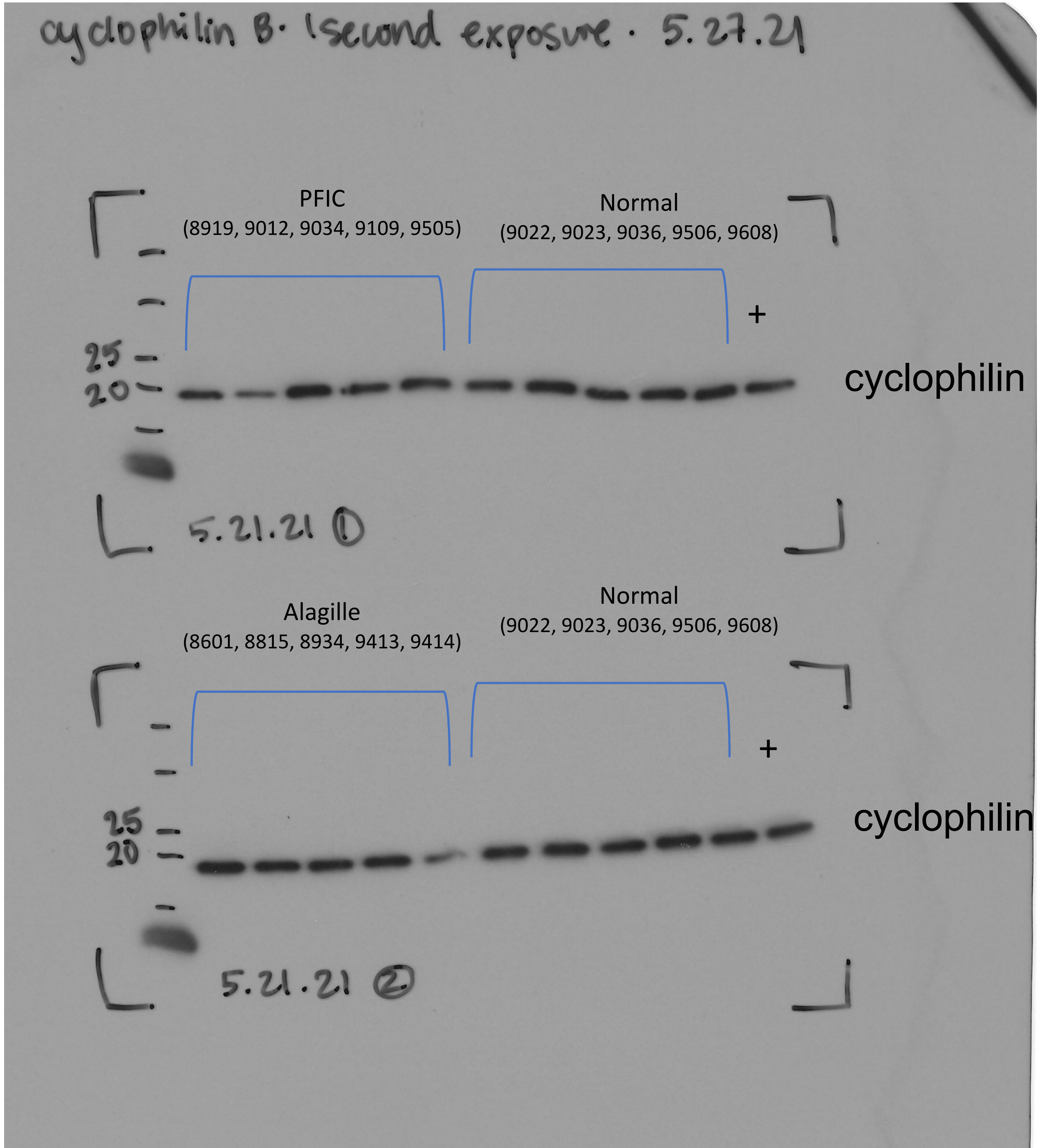

Supplement: S1 Raw images — (PDF) [file pone.0279016.s009.pdf]
